# Integrative Analysis of Methylome and Transcriptome Reveals the Regulatory Mechanisms of Hair Follicle Morphogenesis in Cashmere Goat

**DOI:** 10.3390/cells9040969

**Published:** 2020-04-14

**Authors:** Shanhe Wang, Fang Li, Jinwang Liu, Yuelang Zhang, Yujie Zheng, Wei Ge, Lei Qu, Xin Wang

**Affiliations:** 1 College of Animal Science & Technology, Northwest A&F University, Yangling 712100, China; shanhe@nwsuaf.edu.cn (S.W.); fangli0909@nwsuaf.edu.cn (F.L.); zhangyuelang@nwafu.edu.cn (Y.Z.); Zhengyujie@nwafu.edu.cn (Y.Z.); gewei0901@nwsuaf.edu.cn (W.G.); 2 College of Animal Science & Technology, Yangzhou University, Yangzhou 225000, China; 3 College of Life Science, Yulin University, Yulin 712100, China; ljw_yl@163.com

**Keywords:** hair follicle morphogenesis, differentiation, DNA methylation, lncRNA, cashmere goat, Wnt signaling

## Abstract

Studies in humans and mice have revealed that hair follicle morphogenesis relies on tightly coordinated ectodermal–mesodermal interactions, involving multiple signals and regulatory factors. DNA methylation and long non-coding RNA (lncRNA) play a critical role in early embryonic skin development by controlling gene expression. Acting as an indirect regulator, lncRNA could recruit DNA methyltransferases to specific genomic sites to methylate DNA. However, the molecular regulation mechanisms underlying hair follicle morphogenesis is unclear in cashmere goat. In this study, RNA-seq and whole-genome bisulfite sequencing (WGBS) in embryonic day 65 (E 65) and E 120 skin tissues of cashmere goat were used to reveal this complex regulatory process. The RNA-seq, qRT-PCR, and immunohistochemistry results showed that Wnt signaling played an important role in both hair follicle induction and differentiation stage; transcriptional factors (TFs), including HOXC13, SOX9, SOX21, JUNB, LHX2, VDR, and GATA3, participated in hair follicle differentiation via specific expression at E 120. Subsequently, the combination of WGBS and RNA-seq analysis showed that the expression of some hair follicle differentiation genes and TF genes were negatively correlated with the DNA methylation level generally. A portion of hair follicle differentiation genes were methylated and repressed in the hair follicle induction stage but were subsequently demethylated and expressed during the hair follicle differentiation stage, suggesting that DNA methylation plays an important role in hair morphogenesis by regulating associated gene expression. Furthermore, 45 upregulated and 147 downregulated lncRNAs in E 120 compared with E 65 were identified by lncRNA mapping, and then the potential differentially expressed lncRNAs associated with DNA methylation on the target gene were revealed. In conclusion, critical signals and genes were revealed during hair follicle morphogenesis in the cashmere goat. In this process, DNA methylation was lower in the hair follicle differentiation compared with the hair follicle induction stage and may play an important role in hair morphogenesis by regulating associated gene expression. Furthermore, potential lncRNAs associated with DNA methylation on target genes were delineated. This study enriches the regulatory network and molecular mechanisms on hair morphogenesis.

## 1. Introduction

Hair is a primary characteristic of mammals, and exerts a wide range of functions, including thermoregulation, physical protection, sensory activity, and social interactions [1,2]. Cashmere is an upmarket textile material produced by the secondary hair follicle with high economic value [3,4]. As the number and quality of cashmere depend on cashmere morphogenesis, it is therefore of great value to dissect the critical genes, signaling pathways, and their regulatory machinery underlying hair follicle morphogenesis in the cashmere goat.

Hair follicle morphogenesis takes place during embryonic skin development, which relies on tightly coordinated ectodermal–mesodermal interactions [5,6,7,8]. Researches in mice showed that hair follicle morphogenesis is initiated after secreted epidermal Wnts activate broad dermal Wnt signaling [9], which in turn, through unknown dermal signaling and subsequent Wnt, Eda, and Fgf20 epidermal downstream signaling, leads to hair placode (Pc) induction in the epidermis [2,10,11] and dermal condensate (DC) formation below [12,13]. Following the induction stage, hair follicles enter organogenesis and the subsequent cytodifferentiation stage, in which Pc cells give rise to all the epithelial components of a fully developed hair follicle, including the outer root sheath, inner root sheath, hair matrix, hair shaft, and hair follicle stem cell, while DC cells develop into the follicular dermal papilla and connective tissue sheath [14,15,16]. A previous morphology study on the Inner Mongolia cashmere goat showed that cashmere hair follicle induction was initiated around embryonic day 65 (E 65), and subsequent differentiation thrived around E 120 [17]. A number of molecules and their interactions in each phase, which play a role in hair follicle development, have been identified using the transgenic mice model and hair follicle regeneration assay [18,19,20]. However, the unique molecular features of specific cell types and the regulatory relationships between the signaling pathways involved in these processes are largely unknown [21], especially in cashmere.

Hair follicle morphogenesis results from the process of temporal-spatial expression of genes under the control of genetic and epigenetics, while DNA methylation has been shown to be implicated in the regulation of cell- or tissue-specific gene expression during embryogenesis [22,23]. DNA methylation undergoes dynamic remodeling during early embryogenesis to initially establish a globally demethylated state and then, subsequently, a progressively lineage-specific methylome that maintains the cellular identity and genomic stability [24,25]. As development and differentiation proceed, differentiated cells accumulate epigenetic marks that differ from those of pluripotent cells, and differentiated cells of different lineages also accumulate different marks [26,27]. Corresponding with that, Bock revealed that DNA methylation changes were locus specific and frequently overlapped with lineage-associated transcription factors, and their binding sites. *CEBPB*, *GATA3*, and *HOXA5*, were under the control of DNA methylation and involved in skin and hair follicle differentiation [28]. Through integrated analysis of the methylome and transcriptome, Xiao found 14 crucial factors for wool fiber development under the control of epigenetic mechanisms during curly fleece dynamics in Zhongwei goats [29]. Li revealed that FMN1, PCOLCE, SPTLC3, and COL5A1 were crucial factors for elucidating the epigenetic mechanisms contributing to the telogen-to-anagen transition in cashmere goats [30]. In mice, Li demonstrated that DNA methylation played an important role in maintaining hair follicle stem cells’ homeostasis during development and regeneration [31]. However, the function of DNA methylation in regulating cell lineage specification during hair morphogenesis is still unknown.

DNA methyltransferases (DNMTs) involved in DNA methylation lack sequence-specific DNA binding motifs, while many long non-coding RNAs (lncRNAs) have DNA- and protein-binding motifs, allowing them to carry DNMTs to specific genomic sites [32]. LncRNA-HIT functions as an epigenetic regulator of chondrogenesis by recruiting of p100/CBP complexes [33]. LncRNA-LBCS inhibits the self-renewal and chemoresistance of bladder cancer stem cells through epigenetic silencing of SOX2 [34]. These data indicate that lncRNAs function as guides and tethers, and may be the molecules of choice for epigenetic regulation. Meanwhile, previous study revealed that lncRNA5532 regulates human hair follicle stem cell proliferation and differentiation [35]. However, whether lncRNAs mediate DNA methylation and contribute to hair morphogenesis in the cashmere goat is unknown. 

To investigate the critical genes, signaling pathways, and regulatory mechanism underlying hair morphogenesis in the cashmere goat, RNA-seq, lncRNA mapping, and whole genome bisulfite sequencing (WGBS) were conducted on E 65 and E 120 skin samples. Altered expression patterns of messenger RNAs (mRNAS) and lncRNAs as well as genome-wide DNA methylation profiles were revealed. Furthermore, several signaling pathways and transcriptional factors (TFs) were identified as participating in hair follicle induction and differentiation. Through integrated analysis of the mRNA and lncRNA transcriptome with WGBS data, the regulation of DNA methylation on hair induction and differentiation and the potential lncRNAs involved in DNA methylation taking part in hair morphogenesis were delineated. Our work enriches the underlying molecular mechanisms of hair follicle morphogenesis and skin development.

## 2. Materials and Methods

### 2.1. Animals

Shanbei White Cashmere goats from Shanbei cashmere goats engineering technology research center of Shaanxi Province in China were used in this study. The experimental animals were healthy and under the same management. According to the previous morphology of a study on hair morphogenesis of cashmere goats [17], six pregnant Shanbei White Cashmere goats (two years old, weighing 30–40 kg) were selected to obtain fetal skin samples at E 65 and E 120. Each developmental stage had three replicates. Skin samples were obtained as we previously described [36]. At the same time, other tissues, including muscle, adipose, heart, liver, spleen, lungs, kidney, duodenum, and gonad, were collected. Every tissue sample was divided into two parts: One was fixed with 4% paraformaldehyde and another one was frozen in a sample protector for RNA/DNA (Takara, China) and stored at −80 °C for subsequent analysis.

All the experimental procedures with the goats used in the present study received prior approval from the Experimental Animal Manage Committee of Northwest A&F University (2011-31101684).

### 2.2. Transcriptome Sequencing and Bioinformatics Analysis

Total RNA was extracted from the collected skin and other tissues. The RNA concentration and quality were determined using the Agilent 2100 Bioanalyzer (Agilent Technologies, Santa Clara, CA, USA). To obtain a transcriptome reference of the skin tissue of E 65 and E 120, the skin RNA samples were used to construct RNA-seq libraries from E 65 and E 120. Each developmental stage had three replicates. RNA-seq and subsequent bioinformatics analyses were performed as we previously described [4]. Details are provided in the supplemental experimental procedures (Supplementary Methods).

### 2.3. Quantitative Real-Time PCR (qRT-PCR)

The first-strand cDNA synthesis and qRT-PCR were performed as previously described [36]. Details are provided in the supplemental experimental procedures (Appendix A Additional file 1). Semi-quantitative RT-PCR was performed on a 2720 thermal cycler (Applies Biosystems, Beverly, MA, USA) machine using ES Taq master mix (Cwbio, Beijing, China). The primers used are provided in Table S1.

The primers’ efficiency, including target genes and the reference gene, was calculated using the standard curve and met the criterion of 95–105%. Differences between E 65 and E 120 samples were calculated based on the 2^−ΔΔCt^ method and normalized to β-actin. Each stage included three biological replicates and all samples were run in triplicate. Differences in gene expression between the groups were detected by an independent sample *t*-test.

### 2.4. Histology and Immunohistochemistry (IHC)

Skin samples from E 65 and E 120 were fixed with 4% paraformaldehyde, followed by dehydration further embedded in paraffin and cut into 5-µm sections with a microtome (Leica RM2255, Nussloch, Germany). Sections were rehydrated, blocked with 10% goat serum and 3% bovine serum albumin (Merck KGaA, Darmstadt, Germany), and incubated for 40 min at room temperature. Primary antibody against interest protein was then incubated with the samples at 4 °C overnight. The primary antibodies used were: BMP2 (Abcam, Shanghai, China, Cat. No. ab214821, rabbit 1:200), SOX9 (Abcam, Shanghai, China, Cat. No. ab185966, rabbit 1:200), VDR (Proteintech, Rosemont, IL, USA, Cat. No. 14526-1-AP, rabbit 1:150), SOX2 (Proteintech, Cat. No. 11064-1-AP, rabbit 1:150), BMP4 (Proteintech, Cat. No. 12492-1-AP, rabbit 1:150), β-catenin (Proteintech, Cat. No. 51067-2-AP, rabbit 1:150), WLS (Proteintech, Cat. No. 17950-1-AP, rabbit 1:100), FZD10 (Proteintech, Philadelphia, USA, Cat. No. 18175-1-AP, rabbit 1:150), EDAR (Sangon Biotech, Shanghai, China, Cat. No. D160287, rabbit 1:100), and FGF20 (Sangon Biotech, Shanghai, China, Cat. No. D161681, rabbit 1:100). Subsequently, fluorescent goat anti-rabbit Ig-CY3/FITC-conjugated secondary antibody (Beyotime Biotechnology, Shanghai, China, Cat. No. A0516/A0562, goat, 1:100) or HRP-conjugated secondary antibody (Sangon Biotech, Cat. No. 110058, goat, 1:100) were used to specifically bind to the primary antibody. A Metal Enhanced DAB Substrate Kit (Solarbio, Beijing, China) was used for color development under the catalysis of HRP. Hoechst33342 (Beyotime Biotechnology, Shanghai, China) was used for nuclei staining and the slides were finally mounted with Vecatshield mounting media (VECTOR, Burlingame, CA, USA). Hematoxylin and eosin (H&E) staining was performed according to standard procedures. Fluorescent pictures were taken under a LEICA TCS SP5 II confocal microscopy (Leica Microsystems GmbH, Wetzlar, Germany). All images of H&E-stained sections were taken on an Eclipse 80i microscope (Nikon, Tokyo, Japan). 

### 2.5. DNA Extraction, WGBS Library Construction, and Sequencing

Genomic DNA was extracted from skin samples (E 65 and E 120) using a Qiagen DNeasy Blood & Tissue Kit (Qiagen, Germantown, MD, USA) according to the manufacturer’s instructions. Genomic DNA degradation and contamination were monitored on agarose gels. DNA purity and concentration were checked using the NanoPhotometer^®^ spectrophotometer (IMPLEN Gmbh, Munich, Germany).

WGBS was performed as previously described [30] in the E 65 and E 120 skin tissues of cashmere goats. Each developmental stage had three biological replicates. A total of 5.2 μg of genomic DNA spiked with 26 ng lambda DNA were fragmented by sonication to 200–300 bp with Covaris S220, followed by end repair and adenylation. Cytosine-methylated barcodes were ligated to sonicated DNA according to the manufacturer’s instructions. Then, these DNA fragments were treated twice with bisulfite using an EZ DNA Methylation-GoldTM Kit (Zymo Research, California, USA), before the resulting single-strand DNA fragments were PCR amplificated using KAPA HiFi HotStart Uracil + ReadyMix (2X). The library concentration was quantified by Qubit^®^ 2.0 Flurometer (Life Technologies) and quantitative PCR, and the insert size was assayed on an Agilent Bioanalyzer 2100 system.

The libraries were sequenced on an Illumina Hiseq 4000 platform and 150-bp paired-end reads were generated. Image analysis and base calling were performed with an Illumina CASAVA pipeline. We used FastQC (fastqc_v0.11.5) to perform basic statistics on the quality of the raw reads. Then, the reads sequences produced by the Illumina pipleline in FASTQ format were pre-processed through Trimmomatic (Trimmomatic-0.36) software using the parameter (SLIDINGWINDOW: 4:15; LEADING:3, TRAILING:3; ILLUMINACLIP: adapter.fa: 2: 30: 10; MINLEN:36). The remaining reads that passed all the filtering steps were counted as clean reads and all subsequent analyses were based on this.

### 2.6. Date Analysis, Identification of DMRs, and Functional Enrichment Analysis

Read mapping, methylation site identification, and differentially methylated analysis were performed as previously described [30]. Details are provided in the supplemental experimental procedures (Supplementary Methods). According to the distribution of DMRs through the genome, we defined the genes related to DMRs as genes whose gene body region (from TSS to TES) or promoter region (upstream 2 kb from the TSS) had an overlap with the DMRs. GO enrichment and KEGG pathway analyses were conducted for the differentially methylated and expressed genes to investigate their biological processes and functions.

### 2.7. Bisulfite Sequencing Polymerase Chain Reaction (BSP)

BSP was performed as we previously described [36] using E 65 and E 120 skin tissues genomic DNA. Every stage included three biological repetitions. We sequenced at least 5 clones for an individual; hence, there were more than 15 clones for one specific DMR at each stage. Online QUMA software was used to process the final sequencing results. The PCR primer sequences used for amplifying the targeted products are shown Table S1. Further details and the primers used are provided in the supplemental experimental procedures (Supplementary Methods).

## 3. Results

### 3.1. The Morphology of Hair Follicle Induction and Differentiation Stages in Cashmere Goats

**Figure 1:**
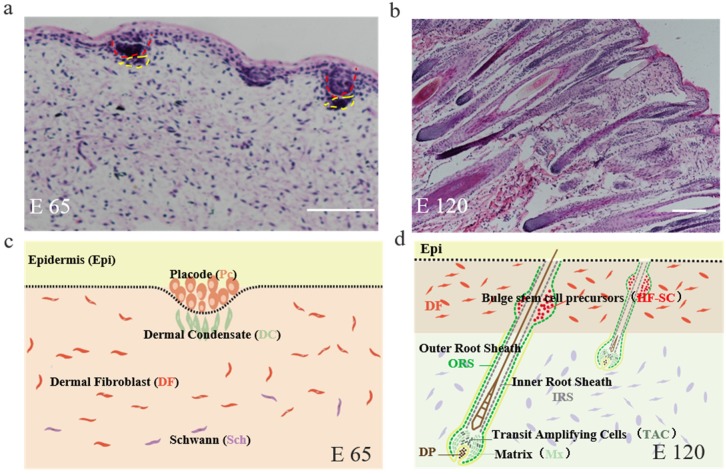
The skin morphology of embryonic day 65 (E 65) and E 120 during hair morphogenesis in Shanbei White Cashmere Goat. (**a**,**b**) The skin morphology of E 65 and E 120 during hair morphogenesis detected by hematoxylin and eosin staining (scale bars, 50 μm); (**c**,**d**) Schematic diagram of the skin morphology in E 65 and E 120 in Shanbei White Cashmere goat. E 65 represents the hair follicle induction stage in which the hair placode (Pc) and dermal condensate (DC) are forming, E 120 represents the hair differentiation stage in which the majority of primary hair follicles mature with a complete structure and the hair shafts emerge through the epidermis, while the hair canal of the secondary hair follicle is visible and the hair shaft begins to grow upwards. In this process, Pc cells give rise to all the epithelial components of fully developed hair follicles, including the outer root sheath, inner root sheath, hair matrix, hair shaft, and hair follicle stem cell, while the DC cells develop into the follicular dermal papilla and connective tissue sheath. Red dashed lines indicate the epidermal hair follicle placode; yellow dashed lines indicate the dermal condensate.

Firstly, the corresponding hair follicle morphogenetic stages form E 65 and E 120 fetus cashmere skin were identified by H&E staining. It revealed that the hair follicle morphogenesis of cashmere goat was initiated around E 65 with the characteristics of crowded epidermal keratinocytes, which were shown as enlarged and elongated, and became organized as a microscopically recognizable hair placode (Pc). Meanwhile, Pc formation was succeeded along with the dermal condensate (DC) of specialized fibroblasts in the underlying mesenchyme (Figure 1a,c). Up to E 120, the majority of primary hair follicles had matured with a complete structure and a hair shaft had emerged through the epidermis, while the hair canal of the secondary hair follicle was visible and the hair shaft began to grow upwards (Figure 1b,d). In general, E 65 represented the induction stage, while E 120 represented the differentiation stage of hair follicle morphogenesis.

### 3.2. Defining Distinct Molecular Signatures of Hair Follicle Induction and Differentiation 

**Figure 2:**
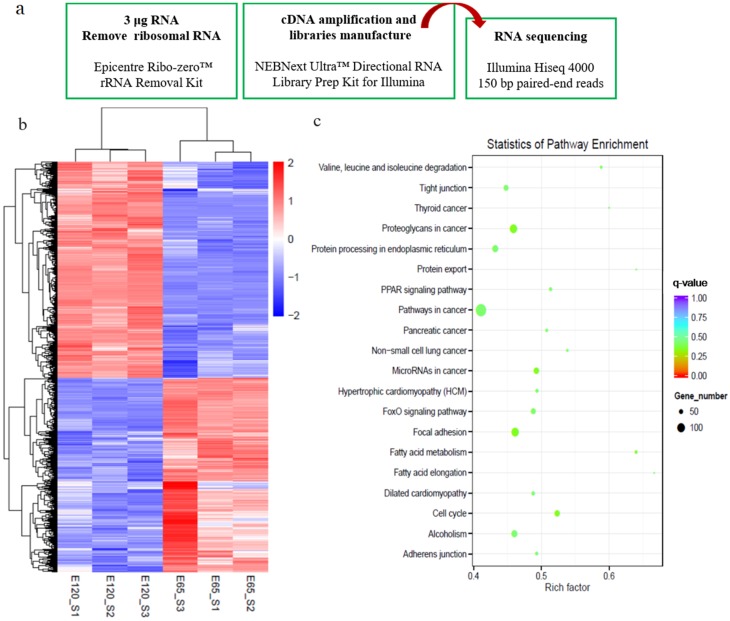
Differential expression genes (DEGs) and critical signals for hair follicle induction and differentiation stages were revealed by RNA-seq. (**a**) Workflow of the sample preparation for RNA-seq. (**b**) The heatmap of DEGs between E 65 and E 120. (**c**) Kyoto Encyclopedia of Genes and Genomes (KEGG) analysis of DEGs between E 65 and E 120. In total, 1729 downregulated genes and 1937 upregulated genes were identified in E 120 compared with E 65.

To reveal the distinct molecular signatures underlying hair follicle induction and differentiation in cashmere goat, we performed RNA-seq on E 65 and E 120 skin tissues using an Illumina Hiseq 4000 system (Figure 2a). This approach resulted in a high-quality output of about 94.9% index reads with a quality score (*q* score) > 30 for all samples. On average, 99 million total clean reads and 93 million aligned reads were produced per sample. Next, we proceeded by mapping, aligning, and quantifying these reads to compute differentially expressed genes between the E 65 and E 120 stages.

**Figure 3:**
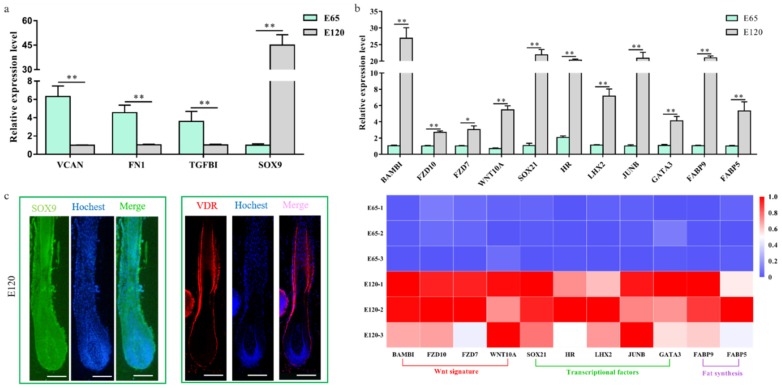
Verification of the differentially expressed genes of hair follicle induction and differentiation (**a**) qRT-PCR of four randomly selected genes between E 65 and E 120 in cashmere goat. (**b**) qRT-PCR confirmed the expression of the partial DEGs associated with hair follicle development between E 65 and E 120 in cashmere goat. Additionally, the heatmap was based on the results of qRT-PCR and standardized by the min-max normalization method. (**c**) Immunofluorescence (IF) of SOX9 and VDR at E 120 of cashmere goat. The expression of specific genes was quantified relative to the expression level of β-actin using the comparative cycle threshold (ΔΔCT) method. The data are expressed as the mean ± SE (*n* = 3). * *p* < 0.05, ** *p* < 0.01. Green/red fluorescence indicated the expression pattern of the interest protein. The nucleus was stained with Hoechst in blue. Scale bars, 50 μm.

By comparing the RNA-seq data between E 65 and E 120, a total of 3666 differentially expressed genes (DEGs, fold change ≥ 2 and *p*-adjust value ≤ 0.05) were found, in which 1729 genes were downregulated and 1937 genes were upregulated in E 120 compared with E 65 (Figure 2b) (Appendix A Additional file 1). KEGG analysis of the DEGs revealed significant functional enrichment of cell migration and aggregation, highlighting the central roles of intercellular crosstalk and dynamic cell rearrangement in promoting skin and hair follicle development (Figure 2c). Specifically, the Wnt and Eda signaling pathways were enriched in our study, which were previously demonstrated to play an important role in mouse hair induction [9,37]. In addition, enriched Wnt and Notch signaling was demonstrated to take part in mouse hair differentiation [38,39] (Figure S1). To confirm the expression pattern of the DEGs, we randomly selected four genes (*VCAN*, *FN1*, *TGFBI*, *SOX9*) to validate their expression patterns using qRT-PCR (Figure 3a). The results were in accordance with the RNA-seq data, suggesting that the expression patterns based on the RNA-seq data were reliable.

**Figure 4:**
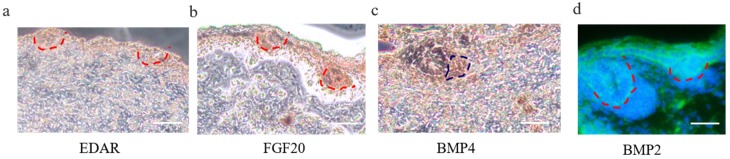
Immunohistochemistry (IHC) verification of Pc and DC cell-type-specific genes on cashmere skin tissue. (**a**–**d**) EDAR, BMP2, and FGF20 are specifically expressed in Pc, while BMP4 is specifically expressed in DC. Brown indicates the expression of the interest protein. Green fluorescence indicates the expression pattern of BMP2; the nucleus was stained with Hoechst in blue. Red dashed lines indicate the epidermal hair follicle placode; blue dashed lines indicate the dermal condensate. Scale bars, 50 μm.

We revealed that a number of keratin and keratin-associated protein genes were upregulated or specifically expressed in E 120 (Appendix A Additional file 1), which was in accordance with the phenotype of hair shaft development in E 120 and that keratin and keratin-associated protein are major structural proteins of the hair shaft [40]. Correspondingly, signaling genes belonging to the Wnt and Notch pathways were upregulated in E 120; at the same time, transcriptional factors with an established role in mice hair follicle differentiation, including HOXC13, SOX9, SOX21, JUNB, LHX2, VDR, DLX3, and GATA3 [41,42,43,44,45,46,47], were upregulated or specifically expressed in E 120, as detected by RNA-seq (Appendix A Additional file 1), qRT-PCR (Figure 3b), and semi-quantitative RT-PCR (Figure S2). Furthermore, the expression of SOX9 and VDR was reconfirmed using immunofluorescence (IF). The results showed that SOX9 was mainly expressed in the outer root sheath and VDR mainly expressed in the outer root sheath and hair shaft in E 120 (Figure 3c) while it was not expressed in E 65 (data not shown). These results highlight the central roles of these transcriptional factors and signals in hair follicle differentiation. Besides, we found several specific genes, which were the critical genes in specific cell types (Pc and DC) during hair follicle morphogenesis at E 14.5 in mice [8,21,48], were expressed at E 65 of cashmere goat (FPKM > 0.5) (Figure S3), which indicated that these genes may play important roles in hair induction. To further validate the specificity of these genes, we performed IHC validation. The result showed that EDAR, BMP2, and FGF20 were specifically expressed in Pc, while BMP4 was specifically expressed in DC (Figure 4a–d), which suggests that these genes could be markers for Pc and DC of cashmere goat.

### 3.3. Wnt Signal in Hair Follicle Induction and Differentiation

**Figure 5:**
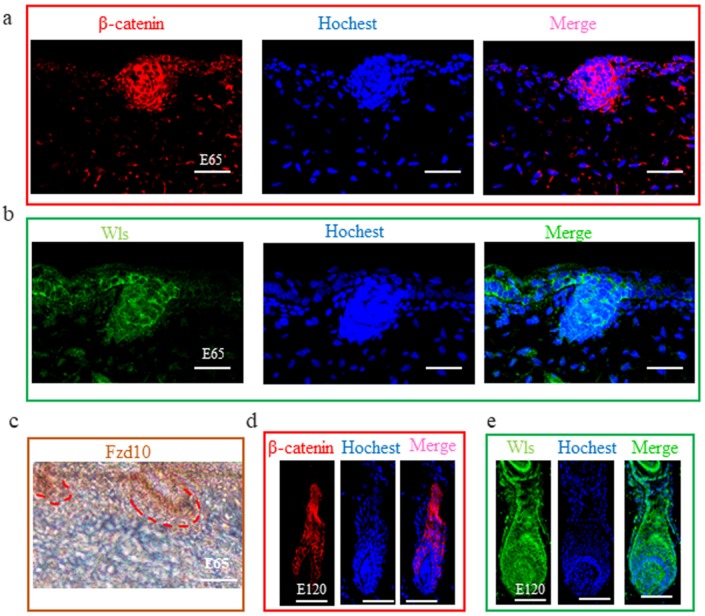
The expression of β-catenin, WLS, and FZD10 at hair follicle induction and differentiation stages were detected by IHC. (**a**–**c**) The expression of β-catenin, WLS, and FZD10 at the E 65 stage. (**d**,**e**) The expression of β-catenin and WLS at the E 120 stage. Both β-catenin and WLS were expressed in the epidermal hair placode at E 65 stage, suggesting that Wnt signals in the hair placode were activated under the control of the Wnt ligand from the hair placode. At E 120, β-catenin and WLS were expressed in the outer root sheath, matrix, and hair shaft. Green/red fluorescence indicated the expression pattern of the interest protein. The nucleus was stained with Hoechst in blue. Brown indicates the expression of the FZD10 protein. Red dashed lines indicate the epidermal hair follicle placode. Scale bars, 50 μm.

From our study and previous studies, Wnt signaling is one of the foremost signaling during hair induction and hair differentiation [9,18]. However, which cell generates the Wnt signal molecules and which cell receives the signal during hair induction is still unclear. β-catenin is stabilized and expressed in the nucleus when extracellular Wnt proteins bind to frizzled receptors and low-density-related lipoproteins in the target cell’s membrane [49]. Hence, in our study, we detected the expression of β-catenin using IF to reflect the activated Wnt signal. The result revealed that β-catenin was expressed in the epidermal hair follicle placode (Figure 5a), suggesting the Wnt signal is activated in epidermal cells during hair induction. Consistent with this, FZD10, the receptor of Wnt ligands, was also expressed in the epidermal hair follicle placode (Figure 5c). Meanwhile, Wnt ligands are lipid-modified extracellular glycoproteins that require the activity of Wntless protein (WLS) for secretion [50]. In order to investigate which cell emits the Wnt ligands, the expression of WLS protein by IF on dorsal skin at E 65 was examined. WLS protein was detectable in the surface ectoderm as cytoplasmic staining and was enriched in the early developing hair follicle placode rather than dermal cells (Figure 5b). This result suggests that the Wnt signal in the hair placode is activated under the control of the Wnt ligand from the hair placode. At E 120, WLS and β-catenin were expressed in the outer root sheath, matrix, and hair shaft (Figure 5d,e), which was in accordance with previous studies in mice [51], suggesting that the Wnt signal also plays an important role in cashmere goat hair differentiation.

### 3.4. LncRNA Analysis of Skin Hair Follicle Development

**Figure 6:**
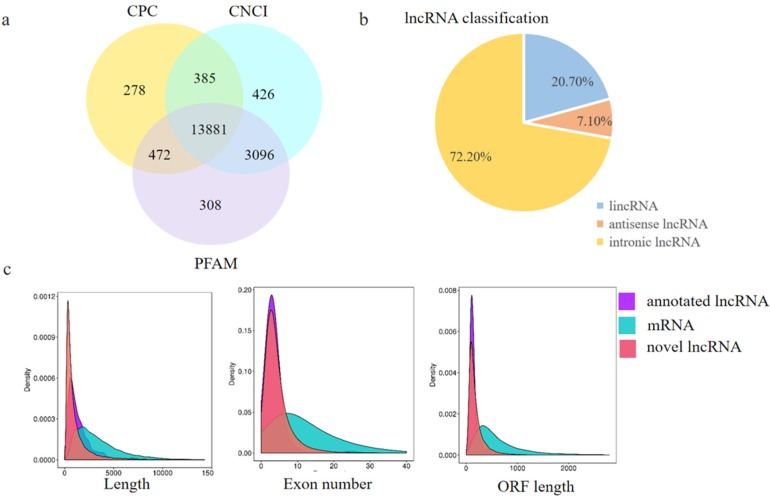
Identification and characterization of lncRNAs in E 65 and E 120 skin tissues of *Capra hircus*. (**a**) Screening of the candidate lncRNAs in the skin transcriptome by CPC, CNCI, and PFAM. (**b**) The classification of lncRNAs. (**c**) Distribution of the transcript lengths, exon number, and open reading frame (ORF) length in the lncRNAs and protein-coding transcripts. In total, 1407 annotated lncRNAs and 13,881 novel lncRNA loci, including long intergenic non-coding RNA (lincRNAs), intronic lncRNA, and anti-sense lncRNAs, were identified. Compared with protein coding transcripts, lncRNAs showed a shorter ORF length and transcript length, and a lesser exon number.

To investigate whether lncRNA takes part in DNA methylation and plays an important role in hair follicle induction and differentiation, the lncRNA transcriptome from RNA-seq was analyzed to define the lncRNA patterns in E 65 and E 120 skin tissues. After a rigorous process of selection and coding potential analysis using the software CNCI, CPC and Pfam-scan, 1407 annotated lncRNAs (Appendix A Additional file 2) and 13,881 novel lncRNAs loci (Appendix A Additional file 3), including long intergenic non-coding RNA (lincRNAs), intronic lncRNA, and anti-sense lncRNAs, were identified (Figure 6a,b). Compared with protein coding transcripts, lncRNAs showed a shorter open reading frame (ORF) length and transcript length, and a lesser exon number (Figure 6c).

**Figure 7:**
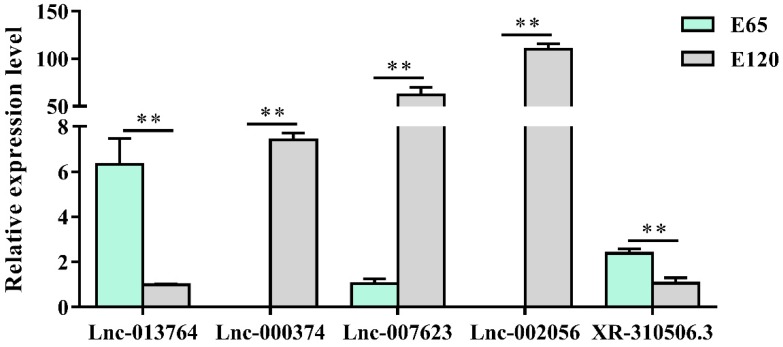
The lncRNA expression patterns in different stages. The results were in accordance with the RNA-seq data and showed that lnc_000374 and lnc_002056 were specifically expressed at E 120. The expression of specific genes was quantified relative to the expression level of β-actin using the comparative cycle threshold (ΔΔCT) method. The data are expressed as the mean ± SE (*n* = 3). ** *p* < 0.01.

Using edgeR, the differentially expressed lncRNAs (fold change ≥ 2 and *p*-adjust value ≤ 0.05) between E 65 and E 120 were screened, resulting in 192 differentially expressed lncRNAs, including 45 upregulated and 147 downregulated lncRNAs in E 120 compared with E 65 (Figure S4a) (Appendix A Additional file 4). Meanwhile, a few lncRNAs were specifically expressed at a single developmental stage of hair morphogenesis, such as lnc_006636, which showed E 65-specific expression, while lnc_000374, lnc_001937 and lnc_009323 showed E 120-specific expression, indicating that these lncRNAs could regulate cashmere morphogenesis through their spatio-temporal expression. Subsequently, we randomly selected five differentially expressed lncRNAs to validate their expression patterns using qRT-PCR. The results were in accordance with the RNA-seq data and showed that lnc_000374 and lnc_002056 were specifically expressed at E 120 (Figure 7), suggesting that the expression patterns based on the RNA-seq data were reliable.

To investigate the function of lncRNAs, the potential targets of lncRNAs in cis and trans were predicted as previously described [4]. Subsequently, KEGG analysis was performed on these target genes. As a result, the target genes were enriched in hair follicle-related signaling pathways, including the Wnt, focal adhesion, and Ecm receptor pathways (Figure S4b), indicating lncRNAs may participate in hair induction and differentiation by regulating related target genes.

### 3.5. Genome DNA Methylation of Hair Induction and Differentiation during Morphogenesis

We found the differential genes between E 65 and E 120, which indicated that hair morphogenesis is a consequence of the spatial and temporal expression of genes. As known, DNA methylation plays a critical role in these genes’ expression [27]. However, the regulation mechanism of DNA methylation during hair morphogenesis remains unknown in cashmere goat. Therefore, we detected the DNA methylation levels of skin tissues at E 65 and E 120 using WGBS. A total of 195.37 G and 187.09 G raw data were generated from the two groups, respectively. An average of 212 million raw reads of WGBS data for the E 65 and E 120 groups were analyzed. Approximately 90.20% (E 65) and 89.6% (E 120) of the clean reads were independently mapped to the goat reference genome assembly ARS1 (Tables S2 and S3). Any ambiguously mapped and duplicate reads were removed from the downstream analysis. Then, the methylation levels of each cytosine were calculated. 

**Figure 8:**
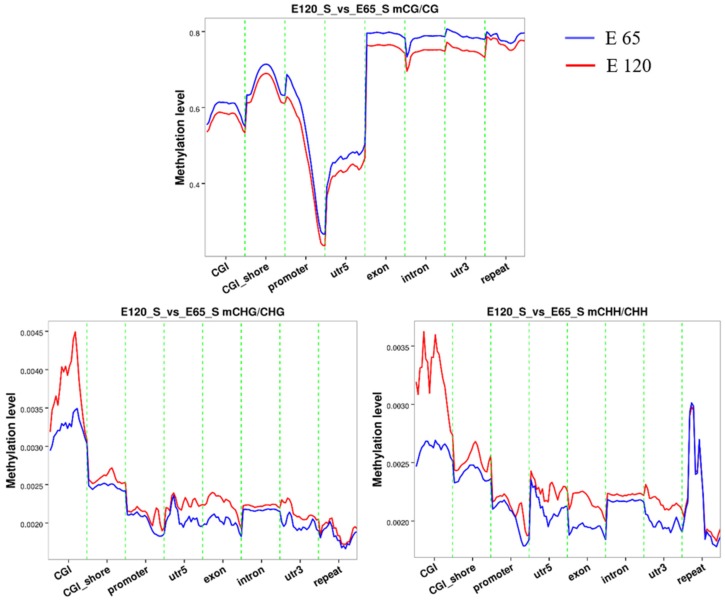
The methylation level in different gene regions for mCG, mCHH, and mCHG. At the genome-wide scale, the E 65 samples exhibited a higher CG methylation status in all regions. Marked hypomethylation was observed in the regions surrounding the transcription start site.

An average of 1.78% and 1.97% methylated cytosines (mCs) of all genomic C sites in E 65 and E 120 were detected, respectively (Table S4), suggesting that the mC level in the hair follicle induction stage was higher than that in the hair follicle differentiation stage during hair follicle morphogenesis. Three classifications of DNA methylation: mCG, mCHH (where H is A, C, or T), and mCHG, were detected in goat samples, in which mCG was the predominant type (> 96%) in both the E 65 and E 120 groups. The methylation levels in different genetic structural regions were determined to examine the overall methylation status, including promoters, exons, introns, CpG islands (CGIs), and CGI shores (regions within 2 kb of an island). As a result, the E 65 samples (hair follicle induction stage) exhibited a higher CG methylation status than E 120 in all regions of the genome-wide scale (Figure 8), which indicates that demethylation took place in E 120 (hair follicle differentiation stage) to ensure the cell lineages. In accordance with this, qRT-PCR showed that TET3, which are intermediates in the process of DNA demethylation as DNA hydroxylases, was expressed higher in E 120 compared with E 65 (Figure S5). Meanwhile, marked hypomethylation was observed in the regions surrounding the transcription start site corresponding with a previous study [52].

**Figure 9:**
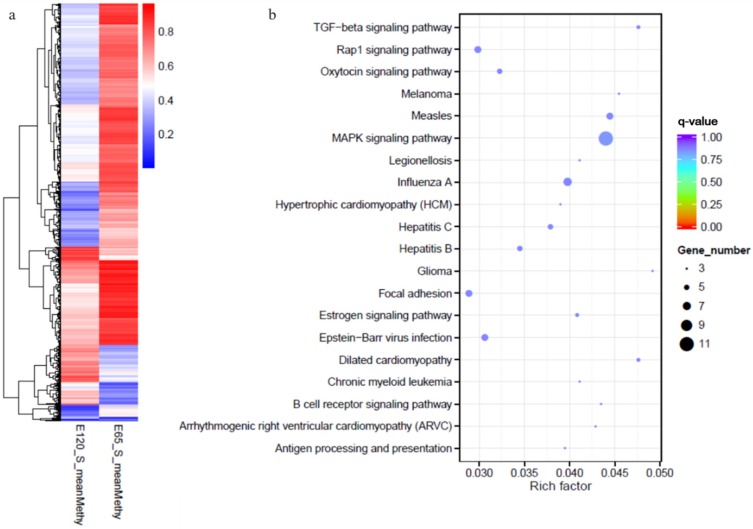
The heat map and Kyoto Encyclopedia of Genes and Genomes (KEGG) analysis of genes with differential methylation between E 65 and E 120. (**a**) The heat map of the genes with differential methylation between E 65 and E 120. (**b**) The KEGG analysis of the genes with differential methylation between E 65 and E 120. In total, 3371 differentially methylated genes (DMGs) were identified between the two stages. KEGG analysis revealed that the DMGs were enriched in TGF-β and focal adhesion signaling pathways.

Subsequently, DSS was used to identify genomic regions with different methylation levels between the E 65 and E 120 stages. A total of 6899 differentially methylated regions (DMRs) were detected, including 5241 hyper DMRs and 1658 hypo DMRs in E 120 compared with E 65 (Appendix A Additional file 5), in which 3371 genes determined to the differentially methylated genes were identified by annotating the DMRs to the goat genome (Figure 9a). To obtain a better understanding of the role of DNA methylation on gene regulatory networks during hair induction and differentiation, the KEGG analysis revealed that the DMGs were enriched in TGF-β and focal adhesion signaling pathways (Figure 9b). These results highlight the central roles of DNA methylation regulation in intercellular crosstalk and signaling transduction during hair follicle induction and differentiation.

### 3.6. Integrated Analysis of WGBS and mRNA-seq Data

**Figure 10:**
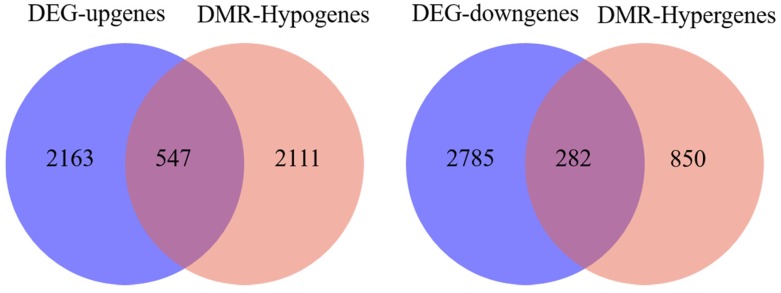
Venn diagram between the differentially methylated genes and differentially expressed genes between E 65 and E 120. In total, 547 hypo-methylation genes had higher expression in E 120 while 282 hyper-methylation genes had lower expression in E 120 compared with E 65.

**Figure 11:**
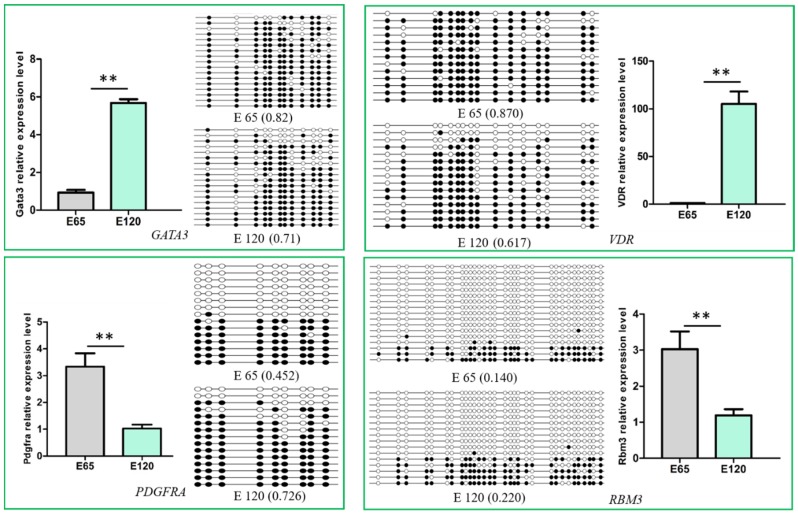
Verification of the differentially methylated genes and their expression. The DNA methylation detected by Bisulfite Sequencing Polymerase Chain Reaction (BSP) was in accordance with the whole-genome bisulfite sequencing (WGBS), and the gene expression was in accordance with the RNA-seq, in which the gene expression was repressed by the high DNA methylation. The expression of specific genes was quantified relative to the expression level of β-actin using the comparative cycle threshold (ΔΔCT) method. The data are expressed as the mean ± 1 SE (*n* = 3). ** *p* < 0.01.

To determine the relationship between DNA methylation and gene expression, the integrated analysis of WGBS and RNA-seq data was performed. As a result, we detected 547 hypo-methylation genes with higher expression while 282 hyper-methylation genes had lower expression in E 120 compared with E 65 (Appendix A Additional file 6) (Figure 10). In order to verify the relationship between DNA methylation and gene expression, four genes involved in hair follicle development were selected to be reconfirmed using BSP and qRT-PCR. The result of the BSP was in accordance with that of the WGBS, and the gene expressions trends were in accordance with the RNA-seq data, in which the genes were repressed by the high DNA methylation (Figure 11). 

It was noteworthy that the transcriptional factor genes associated with hair differentiation, including GATA3, VDR, CUX1, TP63, and RUNX1, had low expression with high DNA methylation during the hair induction stage in our integrated analysis on WGBS and RNA-seq data. Meanwhile, the signaling genes associated with hair differentiation and development, including NOTCH1, NOTCH3, JAG1, FZD1, SMAD7, and keratin gene KRT40, had similar expressions and DNA methylation patterns with the above transcriptional factor genes (Table 1). The results suggest that DNA methylation plays an important role in hair differentiation by regulating associated gene expression. Hair differentiation-related genes were not expressed at the hair induction stage with high methylation, while they were expressed with hypo-methylation during hair differentiation. Demethylation may occur in hair differentiation to regulate DNA methylation and gene expression. 

### 3.7. Potential lncRNA that Could Take Part in DNA Methylation

Furthermore, in order to investigate the function of lncRNAs on gene expression regulation by mediating DNA methylation, an integrated analysis of lncRNAs, the mRNA transcriptome, and WGBS was performed. As a result, the potential differentially expressed lncRNAs associated with DNA methylation on target genes were revealed (Appendix A Additional file 7), such as lncRNA XR_001918556 and lnc-013255, which may affect the DNA methylation of transcriptional factor gene *GATA3* and *TP63*, respectively. Lnc-003786 may affect the signal gene *FGFR2* and lnc-002056 may affect teneurin-2, which encodes transmembrane proteins. Lnc-007623 may affect the DNA methylation of the ADD1 gene, which encodes a cytoskeletal protein. Furthermore, the lncRNA expression patterns in different tissues of E 120 and skin samples of E 65 are shown in Figure S6. We found lnc-002056, lnc-007623, and lnc-000374 were specifically expressed in skin tissue at E 120, corresponding with the hyper DNA methylation of their target genes at E 120, which indicates their potential role in DNA methylation regulation.

## 4. Discussion

Mouse pelage hair follicle formation has been divided into nine distinct developmental stages (0–8) for 20 years [53]. Increasing functional molecules have been identified and characterized for each stage using spontaneous mouse mutants and genetically engineered mice [20,54,55]. However, there are few reports regarding the machinery underlying cashmere goat hair follicle morphogenesis due to technical difficulties and high costs. Although there are conservative signals in hair follicle development among mammals, different physiology and regulation mechanisms exist between mice and cashmere goats. Cashmere is nonmedullated and under the control of the seasonal variation of light, which is different from mice [3,56]. Further evidence of differences is the fact that EDAR gene-targeted cashmere goats show different phenotypes in hair follicles compared with targeted mice [57,58]. As hair follicle morphogenesis and development determine the yield and quality of cashmere, it is critical to reveal the underlying molecular mechanism. Hence, based on the H&E staining results, E 65 and E 120 skin tissues were selected to identify the signals and genes involved in hair induction and differentiation stages.

**Figure 12:**
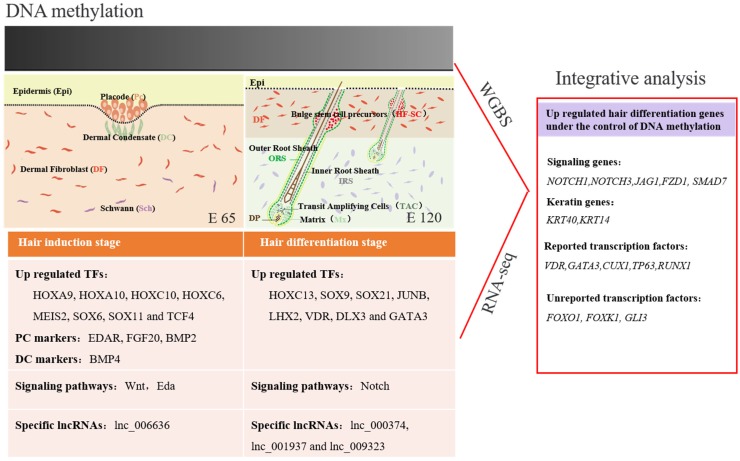
Integrative analysis of the methylome and transcriptome reveals the regulatory mechanisms of hair follicle morphogenesis in Cashmere goat. The darker color represents a higher DNA methylation level.

Hair follicle morphogenesis relies on the interaction between epidermal and dermal cells, ultimately resulting in differentiation of the hair shaft, root sheaths, and dermal papilla [40,55]. Corresponding with this, through RNA-seq and bioinformatics analysis, DEGs were found related to signaling, cell migration, and aggregation, highlighting the central roles of intercellular crosstalk and dynamic cell rearrangement in hair morphogenesis. Specifically, the Wnt signal has been demonstrated to play a critical role in hair induction [59,60]. However, accurate signal transmission between different cells is still unknown during hair induction. Through IF of β-catenin and WLS, we revealed that the Wnt signal in the hair placode is activated under the control of the Wnt ligand from the hair placode. Meanwhile, a number of keratins had a similar expression pattern with some transcriptional factors, which were specifically expressed in E 120, suggesting that these transcriptional factors play critical roles in hair follicle differentiation and keratin expression. Furthermore, the signature genes for Pc and DC were identified through comparison with the related reports on mice [21]. The results illustrated the accurate signal communication between different cells, and could be used as markers to isolate specific cells (Figure 12).

During early embryonic development, cells start from a pluripotent state, from which they can differentiate into multiple cell types, and progressively develop a narrower differentiation potential [61]. Their gene-expression programs become more defined and restricted, in which DNA methylation plays a critical role in this process [22,62]. Unlike embryonic stem cells, progenitors are restricted to a certain lineage but have the potential to differentiate into distinct terminal cell types upon stimulation. During hair morphogenesis, hair progenitor cells start in a multipotent state, from which they can differentiate into many hair cell types, and progressively develop a narrower potential [25,61,63]. However, the DNA methylation changes of lineage-committed progenitors to terminally differentiated cells are largely unknown. Recently, studies have demonstrated that DNA methylation is a critical cell-intrinsic determinant for astrocytes’, muscle satellite cells’, and mammary epithelial cells’ differentiation and development [64]. Sen et al., revealed that the dynamic regulation of DNA methylation patterns was indispensable for progenitor maintenance and self-renewal in mammalian somatic tissue. DNMT1 protein was found enriched in undifferentiated cells, where it was required to retain proliferative stamina and suppress differentiation [65]. However, the change of DNA methylation during hair morphogenesis is still unknown. In our study, we revealed that the level of DNA methylation was lower in the hair follicle differentiation compared with the hair follicle induction stage. Furthermore, hair follicle differentiation genes, including transcriptional factors and signaling genes, were methylated in the hair induction stage but were subsequently de-methylated during differentiation (Figure 12). This result suggests that DNA methylation patterns are required for hair induction and differentiation. Correspondingly, Bock revealed that DNA methylation changes play an important role during in vivo differentiation of adult stem cells [28] and Guo revealed that demethylation events are frequently linked to brain-specific gene activation upon terminal neuronal differentiation [66]. Another related report revealed that DNA methylation had little effect on gene expression during the telogen-to-anagen transition in adult Shanbei White Cashmere goats [30]. It should be noted that the majority of DEGs had little correlation with DNA methylation in our study, which indicates that other regulatory mechanisms, such as histone modification and transcriptional control, may play an imperative role in hair follicle induction and differentiation. The results are in accordance with a previous conclusion that genes required later in development are repressed by histone marks, which confer short-term, and therefore flexible, epigenetic silencing [61].

Above, we revealed that locus-specific DNA methylation changes play a critical role during hair morphogenesis. However, both DNA methyltransferases and polycomb-repressive complexes lack sequence-specific DNA-binding motifs. Increasing evidence indicates that many lncRNAs contain DNA-binding motifs that can bind to DNA by forming RNA:DNA triplexes and recruit chromatin-binding factors to specific genomic sites to methylate DNA and chromatin [67,68]. Besides, lncRNAs have been associated with important cellular processes, such as X-chromosome inactivation, imprinting and maintenance of pluripotency, lineage commitment, and apoptosis [32,69,70]. However, the function of lncRNAs in hair morphogenesis is still unknown. In our study, 45 upregulated and 147 downregulated lncRNAs were identified in E 120 compared with E 65; these lncRNAs may function by targeting hair follicle-related signals and genes. Furthermore, potential lncRNAs involved in DNA methylation were revealed. However, the specific function of lncRNAs needs to be studied further. The results provide a potential regulatory mechanism mediated by lncRNAs during hair morphogenesis.

## 5. Conclusions

The critical signals and genes were revealed during hair follicle morphogenesis in cashmere goat. In this process, DNA methylation was lower in the hair follicle differentiation compared with the hair follicle induction stage, and may play an important role in hair morphogenesis by repressing associated gene expression. Furthermore, potential lncRNAs associated with DNA methylation on the target gene were revealed. This study enriches the regulatory network and molecular mechanisms in hair morphogenesis.

## Figures and Tables

**Table 1:** The genes associated with hair follicle differentiation under the control of DNA methylation.

Gene	E 120 FPKM	E 65 FPKM	Log 2 (Fold Change)	*p*-Value	E 120 Mean Methy	E 65 Mean Methy	Start	End
*TP63*	48.8	5.3	3.20	0.005	0.35	0.70	77226924	77227046
*VDR*	19.3	0.0	Inf	0.000	0.73	0.91	31960999	31961087
*GATA3*	11.1	2.1	2.38	0.005	0.58	0.83	12388542	12388674
*CUX1*	5.0	0.0	11.11	0.011	0.52	0.84	35639445	35639901
*RUNX1*	3.1	1.0	1.59	0.002	0.65	0.19	146939482	146939629
*GLI3*	5.1	3.1	0.72	0.003	0.44	0.88	41410654	41411108
*FOXO1*	11.6	1.4	3.05	0.000	0.40	0.72	64694258	64694508
*FZD1*	18.1	5.8	1.65	0.004	0.33	0.68	111902257	111902552
*NOTCH1*	18.1	6.3	1.53	0.003	0.71	0.86	103351558	103351699
*NOTCH3*	10.6	5.0	1.09	0.017	0.51	0.87	100777692	100777863
*SMAD7*	7.6	1.4	2.42	0.005	0.36	0.80	48827145	48827355
*JAG1*	33.5	8.3	2.02	0.001	0.33	0.79	3752377	3752803
*RORA*	23.5	4.2	2.49	0.003	0.55	0.87	53503152	53503499
*EGFR*	30.8	13.0	1.24	0.004	0.32	0.68	842607	843297
*FGFR2*	3.2	0.9	1.78	0.007	0.44	0.70	10433298	10433462
*KRT40*	28.6	0.1	8.43	0.000	0.39	0.79	40830782	40831215
*KRT14*	1321.9	6.6	7.64	0.000	0.66	0.90	41440140	41440345

Notes: The differently methylated genes were selected according their function; only the genes associated with hair follicle differentiation were selected and shown based on previous reports. FPKM, fragments per kilobase of exon model per million mapped fragments. Methy, relative methylation. start-end, the start and end chromosomal positions of methylation regions. Inf, infinity.

## Supplementary Materials

The following are available online at https://www.mdpi.com/2073-4409/9/4/969/s1, Figure S1: The heatmaps of DEGs associated with signaling pathways related to hair follicle development; Figure S2: Semi-quantitative RT-PCR confirmed the expression of partial DEGs associated with hair follicle development between E 65 and E 120 in cashmere goa; Figure S3: The potential cell-type-specific markers during hair induction and differentiation in Shanbei White Cashmere goat; Figure S4: Differentially expressed lncRNAs and their KEGG analysis in cashmere goat skin between E 65 and E 120 during hair morphogenesis; Figure S5: TET3 was expressed higher in E 120 compared with E 65; Figure S6: The lncRNA expression patterns in different tissues of E 120 and skin different stages; Table S1: Primer list for qRT-PCR; Table S2: Data statistics of WGBS at E 65 and E 120 of cashmere goat; Table S3: The quality control of WGBS data at E 65 and E 120 of cashmere goat; Table S4 The statistics of methylation in genome scale at E 65 and E 120 of cashmere goat; Supplementary Methods: Supplemental experimental procedures.

## Accession Numbers

All the RNA-seq data and WGBS data sets supporting the results of this article have been submitted to the National Center for Biotechnology Information (NCBI) Gene Expression Omnibus (GEO). mRNA-seq data: SAMN13669153, SAMN13669154, SAMN13669155, SAMN13669156, SAMN13669157, SAMN13669158. WGBS-seq data: SAMN13679866, SAMN13679867, SAMN13679868, SAMN13679869, SAMN13679870, SAMN13679871.

## Appendix A

Additional file 1: The differential expressed genes between E 65 and E 120 stages during hair follicle morphogenesis in cashmere goat (xlsx, 4406 KB);

Additional file 2: The sequences of annotated lncRNAs in E 65 and E 120 skin of cashmere goat (xlsx, 2352 KB);

Additional file 3: The sequences of novel lncRNAs in E 65 and E 120 skin of cashmere goat (xlsx, 17465 KB);

Additional file 4: The differential expressed lncRNAs between E 65 and E 120 stages during hair follicle morphogenesis in cashmere goat (xlsx, 69 KB);

Additional file 5: Differentially methylated reg;ions between E 65 and E 120 during hair morphogenesis in cashmere goat (xlsx, 899 KB);

Additional file 6: The DEGs negatively correlated with DNA methylation between E 65 and E 120 of cashmere goat (xlsx, 119 KB);

Additional file 7: The potential differentially expressed lncRNAs associated with DNA methylation on target genes (xlsx, 631 KB).
