# Supplementary material for: Integrative Analysis of Methylome and Transcriptome Reveals the Regulatory Mechanisms of Hair Follicle Morphogenesis in Cashmere Goat
Source: Cells. 2020 Apr 14;9(4):969. doi: 10.3390/cells9040969 (PMC7226977; doi:10.3390/cells9040969)
Supplement: Supplementary file 1 [file cells-09-00969-s001.zip › cells-729398-supplementary/supplementary file/supplementary materials and methods/Supplemental methods.docx]

Integrative analysis of methylome and transcriptome reveals the regulatory mechanisms of hair follicle morphogenesis in Cashmere goat

Shanhe Wang ^1, 2^ ^†^, Fang Li1 ^†^, Jinwang Liu ^3^ Yuelang Zhang^1^, Yujie Zheng^1^, Wei Ge^1^ and Lei Qu^3*^ and Xin Wang^1*^

SUPPLEMENTAL EXPERIMENTAL PROCEDURES

Transcriptome Sequencing and Bioinformatics Analysis

For skin transcriptome sequencing and bioinformatics at E65 and E120, a total amount of 3 μg RNA per sample was used as input material for the RNA library preparations. Firstly, ribosomal RNA was removed using the Epicentre Ribo-zero™ rRNA Removal Kit (Epicentre, USA), and the rRNA was cleaned up by ethanol precipitation. Subsequently, in total six libraries from E65 (*n* = 3) and E120 (*n* = 3) stages were generated from the rRNA-depleted RNA using the NEBNext® Ultra™ Directional RNA Library Prep Kit for Illumina^®^ (NEB, USA) following the manufacturer’s recommendations. Strand-specific sequencing was performed on the Illumina Hiseq 4000, PE 150 system for these libraries (paired-end 100-bp reads). For quality control, raw data were first processed using in-house Perl scripts. In this step, clean data were obtained by trimming reads containing adapter, reads containing over 10*%* of ploy-N, and low-quality reads (> 50% of bases whose Phred scores were < 20) from the raw data. The Phred score [[1](#_ENREF_1)] (Q20, Q30) and GC content of the clean data were calculated. All subsequent analysis was based on the high-quality data.

Then, The high quality reads were mapped independently to the goat genome v2.0 (ftp://ftp.ncbi.nlm.nih.gov/genomes/all/GCA/000/317/765/GCA_000317765.2_CHIR_2.0) using Bowtie v2.0.6 [[2](#_ENREF_2)] and the spliced read aligner TOPHAT v2.0.9 (main parameter: library-type <fr- firststrand >) [[3](#_ENREF_3)]. The mapped reads of each sample were assembled using Cufflinks (v2.1.1) in a reference-based approach [[4](#_ENREF_4)]. Cufflinks was run with‘min-frags-per-transfrag = 0’ and ‘—library-type’, other parameters were set as default. We then adopted five steps to identify goat lncRNAs from the assembled transcripts: (1) transcripts with length < 200 bp were removed; (2) transcripts with exon number < 2 were removed; (3) transcripts were compared with mRNA, rRNA, tRNA, snRNA, snoRNA and pre-miRNA (https://www.ncbi.nlm.nih.gov/) using Cuffcompare v2.1.1 to remove the same or similar transcripts [[4](#_ENREF_4)]. (4) transcripts with FPKM < 0.5 were removed; (5) transcripts that did not pass the protein-coding-score test were removed using the Coding Potential Calculator (CPC) [[5](#_ENREF_5)], PFAM database [[6](#_ENREF_6)] and Coding-Non-Coding Index (CNCI) software [[7](#_ENREF_7)]. CNCI was used with default parameters. For CPC, the NCBI eukaryotes' protein database was used and the e-value was set to ‘1e-10’. For Pfam-scan, each transcript was translated in all three possible frames and Pfam Scan (v1.3) was used to identify the occurrence of any of the known protein family domains documented in the Pfam database (release 27; used both Pfam A and Pfam B) (http://pfam.xfam.org/). Any transcript with a Pfam hit was excluded from the following steps. Pfam searches used default parameters of -E 0.001—domE 0.001.

Cuffdiff (v2.1.1) [[4](#_ENREF_4)] was used to calculate fragments per kb per million reads (FPKM) of both lncRNAs and coding genes in each sample. It was also used to provide statistical routines for determining differential expression in gene expression data using a model based on the negative binomial distribution. Transcripts or genes with a *p*-adjust ≤ 0.05 [[8](#_ENREF_8)] and fold change ≥ 2 were described as differentially expressed between E65 and E120.

To explore the function of lncRNAs, we first predicted the target genes of lncRNAs in cis and trans. The cis role refers to lncRNAs’ action on neighboring target genes. In the present study, the coding genes from 100 kb upstream and downstream of an lncRNA were searched. The trans role refers to the influence of lncRNAs on other genes at the expression level. Pearson’s correlation coefficients were calculated between expression levels of lncRNAs and mRNAs with custom scripts (Pearson correlation ≥ 0.95 or ≤ −0.95). Gene Ontology enrichment analysis of differentially expressed genes was implemented using Gene Ontology Consortium (http://www.geneontology.org/) [[9](#_ENREF_9)]. Gene ontology terms with corrected *p* value less than 0.05 were considered significantly enriched by differentially expressed genes. Pathway analysis was used to identify signiﬁcant pathways for the differentially expressed genes according to the Kyoto Encyclopedia of Genes and Genomes (KEGG) (http://www.genome.jp/kegg/) [[10](#_ENREF_10)]. We used KOBAS software (main parameter: blastx 1e-10; padjust: BH) to test the statistical enrichment of differentially expressed genes in KEGG pathways [[11](#_ENREF_11)].

Quantitative Real-Time PCR (qRT-PCR)

The total RNAs for RNA-seq were also used for quantitative PCR analysis. The first-strand cDNA was obtained using a PrimeScript™ RT reagent Kit with gDNA Eraser (TAKARA, China), and then were subjected to quantification of the mRNAs with β-actin as an endogenous control on the Bio-Rad CFX96 Touch™ Real Time PCR Detection System (Bio-Rad, USA). The qRT-PCR reaction consisted of 10 μL 2 × SYBR^®^ *Premix Ex Taq*™ II (TAKARA, China), 0.8 μL specific forward/reverse primer (10μM), 1 μL cDNA, and ddH_2_O to a final volume of 20 μL. The quantitative PCR was performed using the following conditions: 95 °C for 60 s, 40 cycles of 95 °C for 10 s, and the optimized annealing temperature for 30 s. Semi-quantitative RT-PCR was performed on 2720 thermal cycler (Applies biosystems) machine using ES Taq master mix (Cwbio, China).The primers were listed in table S1.

Gene expression was quantified relative to β-actin expression using comparative cycle threshold (ΔΔCT) method [[12](#_ENREF_12)] through Bio-Rad CFX Manager 3.1 and Microsoft excel 2013. Each stage (E65 and E120) included at least 3 samples, and all reactions were performed in triplicate for each sample. The results are presented as the means ± standard error of the mean (s.e.m.). Differences in gene expression between the groups were detected by [independent sample](javascript:void(0);) *t*-test.

WGBS Library Preparation, Sequencing and Bioinformatics Analysis

For library preparation and quantification, a total amount of 5.2 μg genomic DNA spiked with 26 ng lambda DNA were fragmented by sonication to 200-300bp with Covaris S220, followed by end repair and adenylation. Cytosine-methylated barcodes were ligated to sonicated DNA according manufacturer’s instructions. Then these DNA fragments were treated twice with bisulfite using EZ DNA Methylation-GoldTM Kit (Zymo Research), before the resulting single-strand DNA fragments were PCR amplificated using KAPA HiFi HotStart Uracil + ReadyMix (2X). Library concentration was quantified by Qubit^®^ 2.0 Flurometer (Life Technologies, CA, USA) and quantitative PCR, and the insert size was assayed on Agilent Bioanalyzer 2100 system.

For sequencing, the library preparations were sequenced on an Illumina Hiseq 4000 platform and 150bp paired-end reads were generated. Image analysis and base calling were performed with Illumina CASAVA pipeline. For data quality control, we use FastQC (fastqc_v0.11.5) to perform basic statistics on the quality of the raw reads. Then, those reads sequences produced by the Illumina pipleline in FASTQ format were pre-processed through Trimmomatic (Trimmomatic-0.36) software using the parameter (SLIDINGWINDOW: 4:15; LEADING:3, TRAILING:3; ILLUMINACLIP: adapter.fa: 2: 30: 10; MINLEN:36). The remaining reads that passed all the filtering steps was counted as clean reads and all subsequent analyses were based on this. At last, we use FastQC to perform basic statistics on the quality of clean reads.

For reads mapping to the reference genome, Bismark software (version 0.16.3) [[13](#_ENREF_13)] was used to perform alignments of bisulfite-treated reads to reference genome (-X 700—dovetail). The reference genome was firstly transformed into bisulfite-converted version (C-to-T and G-to-A converted) and then indexed using bowtie2 [[14](#_ENREF_14)]. Sequence reads were also transformed into fully bisulfite-converted versions (C-to-T and G-to-A converted) before they were aligned to similarly converted versions of the genome in a directional manner. Sequence reads that produce a unique best alignment from the two alignment processes (original top and bottom strand) were then compared to the normal genomic sequence and the methylation state of all cytosine positions in the read was inferred. The same reads that aligned to the same regions of genome were regarded as duplicated ones. The sequencing depth and coverage were summarized using deduplicated reads. The results of methylation extractor (bismark_methylation_extractor—no_overlap) were transformed into bigWig format for visualization using IGV browser. The sodium bisulfite non-coversion rate was calculated as the percentage of cytosine sequenced at cytosine reference positions in the lambda genome.

To identify the methylation site, we modeled the sum Mc of methylated counts as a binomial (Bin) random variable with methylation rate r mC~Bln(mC + umC _×_ r). In order to calculate the methylation level of the sequence, we divided the sequence into multiple bins within 10 kb in size. The sum of methylated and unmethylated read counts in each window were calculated. Methylation level (ML) for each window or C site shows the fraction of methylated Cs, and is defined as: ML(C) = reads(mC) / reads(mC) + reads(C). Calculated ML was further corrected with the bisulfite non-conversion rate according to previous studies [[15](#_ENREF_15)]. Given the bisulfite non-conversion rate r, the corrected ML was estimated as: ML(corrected) = ML—r /1—r.

For differentially methylated analysis, differentially methylated regions (DMRs) were identified using the DSS software [[16](#_ENREF_16),[17](#_ENREF_17)], The core of DSS is a new dispersion shrinkage method for estimating the dispersion parameter from Gamma-Poisson or Beta-Binomial distributions. DSS possess three characteristics to detect DMRs. First, spatial correlation. Proper utilization of the information from neighboring Cytosine sites can help improve estimation of methylation levels at each Cytosine site, and hence improve DMR detection. Second, the read depth of the Cytosine sites provides information on precision that can be exploited to improve statistical tests for DMR detection. Finally, the variance among biological replicates provides information necessary for a valid statistical test to detect DMRs, when there is no biological replicate, DSS combining data from nearby Cytosine sites and using them as ‘pseudo-replicates’ to estimate biological variance at specific locations. According to the distribution of DMRs through the genome, we defined the genes related to DMRs as genes whose gene body region (from TSS to TES) or promoter region (upstream 2kb from the TSS) have an overlap with the DMRs.

Bisulphite Sequencing Polymerase Chain Reaction (BSP-PCR)

Genomic DNA of skin samples including different stages (E65 and E120) was extracted following the standard procedures using TIANamp Genomic DNA Kit (Tiangen, China). Every stage included 3 biological repetition. DNA treatment with sodium bisulphite was performed using the EZ DNA Methylation Kit (Zymo Research, USA) according to the manufacturer’s protocol, except that the conversion temperature was changed to 55 °C. The modified DNA samples were diluted in 10 μL of distilled water and should be immediately used in BSP or stored at −80 °C until PCR amplification. The BSP primers were designed by the online MethPrimer software (http://www.urogene.org/methprimer/). The sequences of PCR primers used for amplifying the targeted products were shown Table S1. We used hot start DNA polymerase (Zymo Taq ^TM^ Premix, Zymo Research, USA) for BSP production. PCR was performed in 50 μL of reaction volume, containing 200 ng genomic DNA, 0.3 μM of each primer, Zymo Taq^TM^ Premix 25 μL. The PCR was performed with a DNA Engine Thermal Cycler (Bio- Rad, USA) using the following program: 10 min at 95 °C, followed by 45 cycles of denaturation for 30 s at 94 °C, annealing for 40 s at 52 °C and extension for 30 s at 72 °C, with a final extension at 72 °C for 7 min. The PCR products were gel purified using Gel Purification Kit (Sangon, China), and then subcloned into the pGEM T-easy vector (Promega, USA). Different positive clones for each subject were randomly selected for sequencing (Sangon, China). We sequenced at least 5 clones from each independent set of amplification and cloning, hence, there were more than 15 clones for each DMR at E65 and E120 stage. The final sequence results were processed by online QUMA software15 (http://quma.cdb.riken.jp/top/index.html).

SUPPLEMENTAL REFERENCES

1. Ewing, B.; Hillier, L.D.; Wendl, M.C.; Green, P. Base-calling of automated sequencer traces using PHRED. I. Accuracy assessment. *Genome Res*. **1998**, *8*, 186–194.
2. Langmead, B.; Trapnell, C.; Pop, M.; Salzberg, S. Ultrafast and memory-efficient alignment of short DNA sequences to the human genome. *Genome Boil.* **2009**, *10*, R25.
3. Trapnell, C.; Pachter, L.; Salzberg, S.L. TopHat: discovering splice junctions with RNA-Seq. *Bioinform.* **2009**, *25*, 1105–1111.
4. Trapnell, C.; Roberts, A.; Goff, L.A.; Pertea, G.; Kim, D.; Kelley, D.R.; Pimentel, H.; Salzberg, S.; Rinn, J.L.; Pachter, L. Differential gene and transcript expression analysis of RNA-seq experiments with TopHat and Cufflinks. *Nat. Protoc.* **2012**, *7*, 562–578.
5. Kong, L.; Zhang, Y.-P.; Ye, Z.-Q.; Liu, X.-Q.; Zhao, S.-Q.; Wei, L.; Gao, G. CPC: assess the protein-coding potential of transcripts using sequence features and support vector machine. *Nucleic Acids Res.* **2007**, *35*, W345–W349.
6. Punt, M.; Coggill, P.C.; Eberhardt, R.Y.; Mistry, J.; Tate, J.; Boursnell, C.; Pang, N.; Forslund, K.; Ceric, G.; Clements, J.; et al. The Pfam protein families database*. Nuc. Ac. Res*. **2012**, *40*, D290–D301.
7. Sun, L.; Luo, H.; Bu, D.; Zhao, G.; Yu, K.; Zhang, C.; Liu, Y.; Chen, R.; Zhao, Y. Utilizing sequence intrinsic composition to classify protein-coding and long non-coding transcripts. *Nucleic Acids Res.* **2013**, *41*, e166.
8. Storey, J. The positive false discovery rate: a Bayesian interpretation and the q -value. *Ann. Stat.* **2003**, *31*, 2013–2035.
9. Chibucos, M.C. Gene Ontology Consortium: going forward. *Nuc. Ac. Res*. **2015**, *43*, D1049–1056.
10. Kanehisa, M.; Araki, M.; Goto, S.; Hattori, M.; Hirakawa, M.; Itoh, M.; Katayama, T.; Kawashima, S.; Okuda, S.; Tokimatsu, T.; et al. KEGG for linking genomes to life and the environment. *Nucleic Acids Res.* **2007**, *36*, D480–D484.
11. Mao, X.; Cai, T.; Olyarchuk, J.G.; Wei, L. Automated genome annotation and pathway identification using the KEGG Orthology (KO) as a controlled vocabulary. *Bioinform.* **2005**, *21*, 3787–3793.
12. Schmittgen, T.D.; Livak, K.J. Analyzing real-time PCR data by the comparative C(T) method. *Nat. Protocols* **2008**, *3,* 1101.
13. Krueger, F.; Andrews, S.R. Bismark: a flexible aligner and methylation caller for Bisulfite-Seq applications. *Bioinform.* **2011**, *27*, 1571–1572.
14. Langmead, B.; Salzberg, S. Fast gapped-read alignment with Bowtie 2. *Nat. Methods* **2012**, *9*, 357–359.
15. Lister, R.; Mukamel, E.A.; Nery, J.R.; Urich, M.; Puddifoot, C.A.; Johnson, N.; Lucero, J.; Huang, Y. (Nancy); Dwork, A.J.; Schultz, M.D.; et al. Global Epigenomic Reconfiguration During Mammalian Brain Development. *Sci.* **2013**, *341*, 1237905.
16. Feng, H.; Conneely, K.N.; Wu, H. A Bayesian hierarchical model to detect differentially methylated loci from single nucleotide resolution sequencing data. *Nucleic Acids Res.* **2014**, *42*, e69.
17. Park, Y.; Wu, H. Differential methylation analysis for BS-seq data under general experimental design. *Bioinform.* **2016**, *32*, 1446–1453.
