# Supplementary material for: Integrative Analysis of Methylome and Transcriptome Reveals the Regulatory Mechanisms of Hair Follicle Morphogenesis in Cashmere Goat
Source: Cells. 2020 Apr 14;9(4):969. doi: 10.3390/cells9040969 (PMC7226977; doi:10.3390/cells9040969)
Supplement: Supplementary file 1 [file cells-09-00969-s001.zip › cells-729398-supplementary/supplementary file/supplementary materials and methods/Supplementary Materials.docx]

Regulation of DNA methylation and lncRNA on hair follicle morphogenesis in Cashmere goat (Capra hircus).

Shanhe Wang ^1, 2^, Fang Li ^1^, Jinwang Liu ^3^ Yuelang Zhang^1^, Yujie Zheng^1^, Wei Ge^1^, Lei Qu^3*^ and Xin Wang^1*^

SUPPLEMENTAL FIGURES

**Figure S1**: The heatmaps of DEGs associated with signaling pathways related to hair follicle development

**Figure S2**: Semi-quantitative RT-PCR confirmed the expression of partial DEGs associated with hair follicle development between E 65 and E 120 in cashmere goat

**Figure S3**: The potential cell-type-specific markers during hair induction and differentiation in Shanbei White Cashmere goat

**Figure S4**: Differentially expressed lncRNAs and their KEGG analysis in cashmere goat skin between E 65 and E 120 during hair morphogenesis

**Figure S5**: Tet3 was expressed higher in E 120 compared with E 65

**Figure S6**: The lncRNA expression patterns in different tissues of E 120 and skin different stages

SUPPLEMENTAL TABLES

**Table S1**: Primer list for qRT-PCR

**Table S2**: Data statistics of WGBS at E 65 and E 120 of cashmere goat

**Table S3**: The quality control of WGBS data at E 65 and E 120 of cashmere goat

**Table S4**: The statistics of methylation in genome scale at E 65 and E 120 of cashmere goat

SUPPLMETAL FIGURES


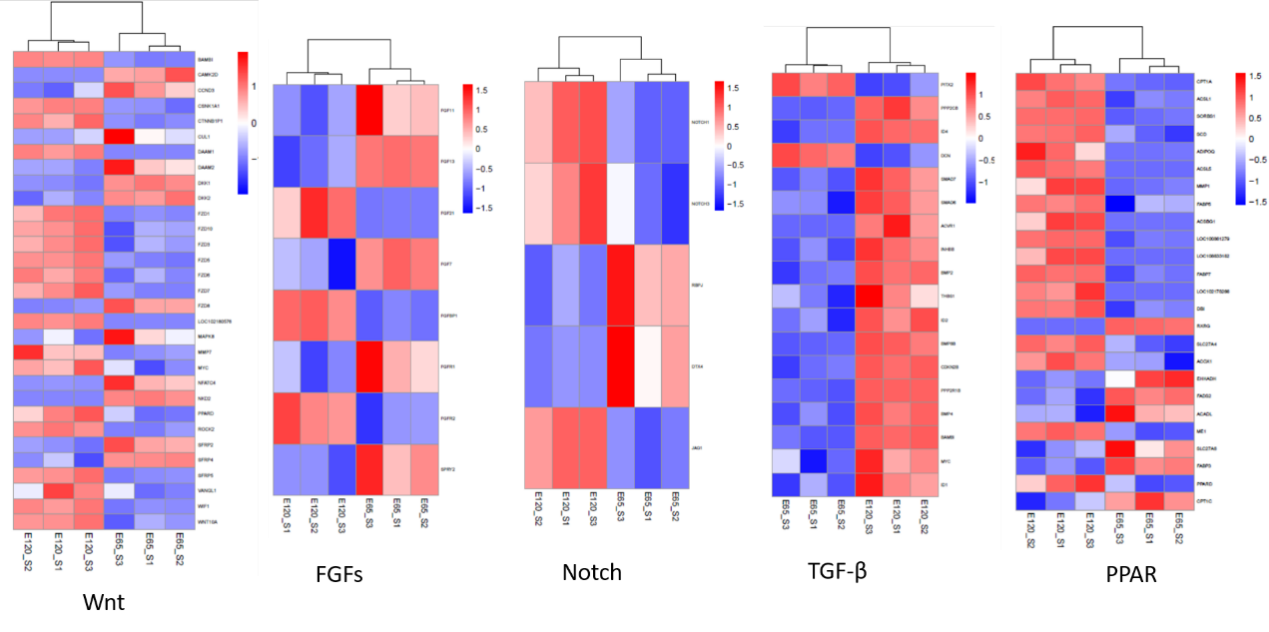


**Figure S1.** The heatmaps of DEGs associated with signaling pathways related to hair follicle development.


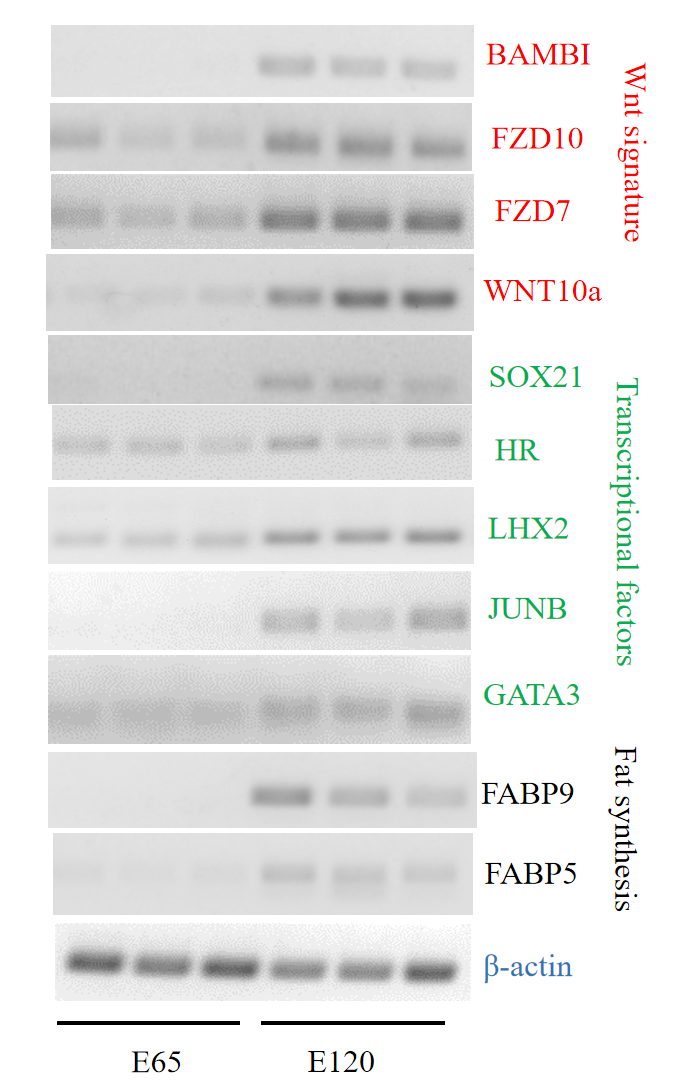


**Figure S2.** Semi-quantitative RT-PCR confirmed the expression of partial DEGs associated with hair follicle development between E 65 and E 120 in cashmere goat. Some previously established transcriptional factors and Wnt signaling genes were up-regulated or specifically expressed in E 120. β-actin was used as reference genes.


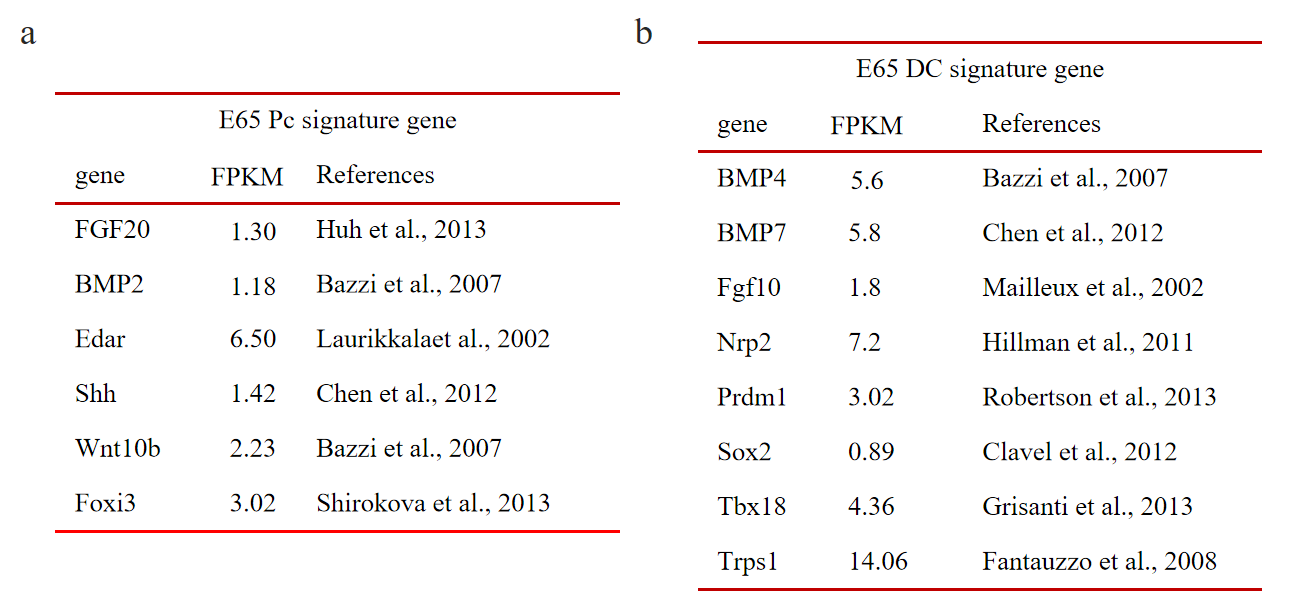


**Figure S3.** The potential cell-type-specific markers during hair induction and differentiation in Shanbei White Cashmere goat. (a) E 65 Pc signature gene. (b) E 65 DC signature gene.


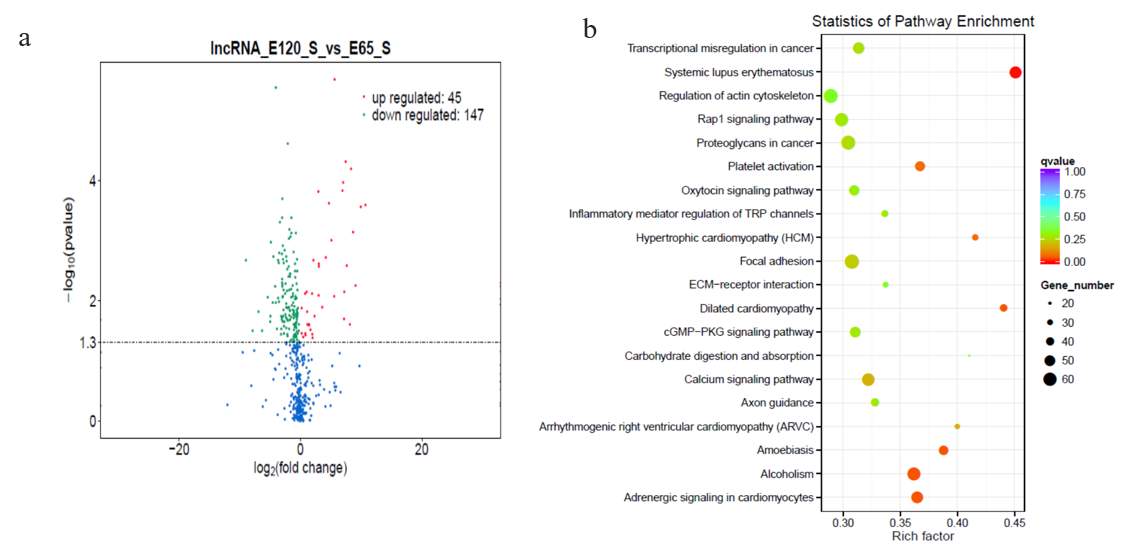


**Figure S4.** Differentially expressed lncRNAs and their KEGG analysis in cashmere goat skin between E 65and E 120 during hair morphogenesis. (a) Differentially expressed lncRNAs. 45 lncRNAs were upregulated, and 147 lncRNAs were downregulated at E 120 compared with E 65. Green dot indicates down regulated lncRNA, red dot indicates up regulated lncRNA at E 120 compared with E 65. (b) KEGG pathways of differentially expressed genes in goat skin between E 65 and E 120. Rich factor indicates the ratio of DEGS enriched in the pathway among genes annotated in the pathway.


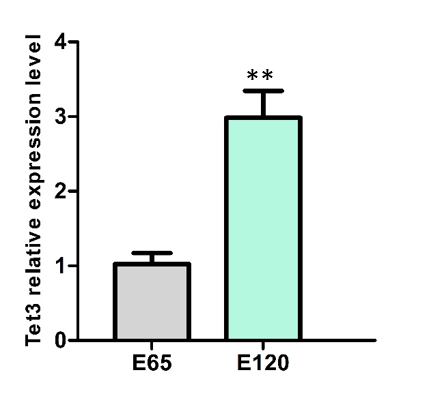


**Figure S5.** Tet3 was expressed higher in E 120 compared with E 65. The data was expressed as the mean ± 1 SE (*n* = 3). ** *p* < 0.01.


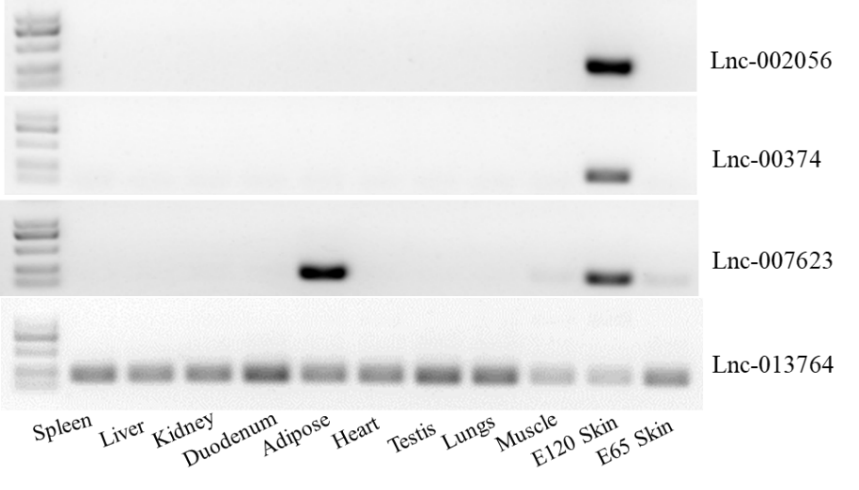


**Figure S6.** The lncRNA expression patterns in different tissues of E 120 and skin different stages. As revealed by semi-quantitative RT-PCR, Lnc-002056, Lnc-007623 and lnc-000374 were specifically expressed in skin tissue at E 120. β-actin was used as reference genes.

SUPPLEMENTAL TABLES

**Table S1.** Primer list for qRT-PCR.

| **Experiment** | **Gene** | **Forward primer** | **Reverse primer** |
| --- | --- | --- | --- |
| qRT-PCR | ACTB | TGAACCCCAAAGCCAACC | AGAGGCGTACAGGGACAGCA |
|  | DKK1 | CTCCAACGCCATCAAGAACACC | GCTGCACTCCGCGTCCTCA |
|  | FZD10 | GAAGACCCCAATCCGCACCA | GAAGACCCCAATCCGCACCA |
|  | FZD7 | TTTATAGGCAAAGCAGCGCAAA | CCTTTAGCCAAGTCAGTACCTC |
|  | BAMBI | TGTCCTCACCCCGCCCAAG | CAGGGCCAGCATAATTAGCAAC |
|  | JUNB | CTGAGGGCGCAACAAACCAC | CCCCTCCCTGTTAAATACACAA |
|  | GATA3 | CTACGTCCCCGAGTACAGC | TTCTGTCCGTTCATCTTGTGGT |
|  | LHX2 | CCATTAACCACAATCCCGATGCC | TGTTTTCCTGCCGTAAAAGGTTGC |
|  | MSX2 | AAAATTCAGAAGACGGAGC | CTTCCGATTGGTCTTGTGT |
|  | SOX21 | CTTTGCACTTCGGAACCTGT | CCGAGAAACTAATGACACCGAT |
|  | FABP5 | CTGTCTGCAACTTTACGGAT | GCAGCTAATTTCCGTGCAA |
|  | FABP9 | AGCCAAGAATCATTATTAGTGC | GTTGTCTCTTTACCAAGCCAT |
|  | TET3 | CTGGAGTCGCCTCTTAAGTACCTG | CCCGTGTAGATGACCTTCTCG |
|  | HR | GCCGGTTCCCTGATGCTCACCC | GGCCTCCATCCCCAGCAGTCCA |
|  | MYOSIN3 | ACAACGCCTATCAGTTCATGCT | ATTCCTCACGGTCTTGGCGTTC |
|  | MYOG | CCAGTGAATGCAGCTCCCATAGCG | CCACTGTGATGCTGTCCACGAT |
|  | MYL7 | CGAGGCTCTTCCAACGTCT | TAGGTCTCTCTAAGGTCCGACT |
|  | RBM3 | GGCAACCTCCCCAGTCTCG | CCTCAGAAATAGGTCCGAAGC |
|  | PDGFRA | ATCCCATTCAATGTTTATGCG | CCTTTGCCCTTCATTTGTCC |
|  | FN1 | CTGAGACCCCAAGTCAACCC | TAAGCTGGCCCTCGTATACCAC |
|  | VDR | CCGGCTTCCATTTCAACGCTA | AGGATCATCTCCCGTTTCCG |
|  | SOX9 | ATGATCGCAGAAAGAACCCAA | GGCCACCAGTCTAACGACA |
|  | VCAN | GCGATGAGAACAGTTACTTACACC | TCACGCCTAGCTTTGTCGTT |
|  | TGFBI | AACTAGCCCCTGTCTATCAGC | CTCATTTCGGTGTGACCCAT |
|  | Lnc-13764 | CCAGAACCCCAACCAGTGCAA | CCCCAGCCCCGAGACCAC |
|  | Lnc-000374 | CTACCCGAGAACAACCTCA | GCCACATCCATAGCCACT |
|  | Lnc-007623 | TATCCCTTGGCTTATGCTCT | ATAAACTTAGATGAAGGTGCTC |
|  | XR31506 | TGTTCCTCATCCGATGTCGTCT | GTCCATTCAATGCCTGTTTGCT |
|  | Lnc-013795 | CCTCTGTCGGCTGCTGTCATTTCG | TGCGTCCAGAGACCCACAGTCCT |
|  | Lnc-002056 | AGTGAAGCACAAAGATCAATCAC | ACACAGGGTAATTTTGTATCCGTA |
|  | Lnc-003786 | CTTCAGCCAAGTTATTTCACGTCA | ATTCCAGGCTCTTTTCTTAGCTC |
| BSP-PCR | RBM3 | TGTTTTTAAGGAATTTAGAGAAAGA | TCCAATAAAACCCAAACCTATATATC |
|  | PDGFRA | AGTAGGTTTTATTGGGTAGGTTTTG | AAACAAATATATAAACAACCCCTTATTC |
|  | GATA3 | GTTAGGATATGAGAGGGTTGTATAG | AAAATATCAAATCAAAACTACCCCA |
|  | VDR | ATTTTAGATAGGGATAGGAAGGTT | CCCTAAACATCTAAACCACCAAA |

**Table S2.** Data statistics of WGBS at E 65 and E 120 of cashmere goat.

| **Sample name** | **Raw Reads** | **Raw Bases(G)** | **Clean Reads** | **Clean Bases(G)** | **Clean ratio (%)** |
| --- | --- | --- | --- | --- | --- |
| E 120_S1 | 213222672 | 63.97 | 211055317 | 62.07 | 97.03 |
| E 120_S2 | 207207656 | 62.16 | 204884499 | 60.18 | 96.81 |
| E 120_S3 | 203193384 | 60.96 | 194484974 | 56.1 | 92.03 |
| E 65_S1 | 213570831 | 64.07 | 211204839 | 62.05 | 96.85 |
| E 65_S2 | 218492151 | 65.55 | 215981411 | 63.45 | 96.8 |
| E 65_S3 | 219165637 | 65.75 | 216545885 | 63.61 | 96.75 |

**Table S3.** The quality control of WGBS data at E 65 and E 120 of cashmere goat.

| **Sample Name** | **Q20 (%)** | **Q30 (%)** | **GC Content (%)** | **BS Conversion rate(%)** | **Mapping rate (%)** | **Duplication rate (%)** |
| --- | --- | --- | --- | --- | --- | --- |
| E 120_S1 | 98.65 | 96.45 | 20.94 | 99.889 | 89.41 | 16.65 |
| E 120_S2 | 98.58 | 96.26 | 20.84 | 99.882 | 89.58 | 16.67 |
| E 120_S3 | 97.82 | 93.81 | 20.95 | 99.892 | 89.68 | 13.13 |
| E 65_S1 | 98.57 | 96.24 | 21.02 | 99.886 | 90.35 | 15.53 |
| E 65_S2 | 98.56 | 96.22 | 21.08 | 99.9 | 90.29 | 16.25 |
| E 65_S3 | 98.56 | 96.22 | 21.03 | 99.881 | 89.98 | 16.44 |

**Table S4.** The statistics of methylation in genome scale at E 65 and E 120 of cashmere goat.

| **Sample name** | **mC percent (%)** | **mCpG percent (%)** | **mCHG percent (%)** | **mCHH percent (%)** |
| --- | --- | --- | --- | --- |
| E 120_S1 | 1.88 | 28.5 | 0.06 | 0.07 |
| E 120_S2 | 1.68 | 25.31 | 0.05 | 0.07 |
| E 120_S3 | 1.78 | 26.92 | 0.06 | 0.08 |
| E 65_S1 | 1.97 | 29.74 | 0.06 | 0.07 |
| E 65_S2 | 1.96 | 29.6. | 0.06 | 0.08 |
| E 65_S3 | 1.98 | 30.00 | 0.06 | 0.07 |
